# Identification of Intermediate-Size Non-Coding RNAs Involved in the UV-Induced DNA Damage Response in *C. elegans*

**DOI:** 10.1371/journal.pone.0048066

**Published:** 2012-11-07

**Authors:** Aqian Li, Guifeng Wei, Yunfei Wang, Ying Zhou, Xian-en Zhang, Lijun Bi, Runsheng Chen

**Affiliations:** 1 Laboratory of Non-coding RNA, Institute of Biophysics, Chinese Academy of Sciences, Beijing, China; 2 Graduate School of the Chinese Academy of Science, Beijing, China; 3 State Key Laboratory of Virology, Wuhan Institute of Virology, Chinese Academy of Sciences, Wuhan, China; Inserm U869, France

## Abstract

**Background:**

A network of DNA damage response (DDR) mechanisms functions coordinately to maintain genome integrity and prevent disease. The Nucleotide Excision Repair (NER) pathway is known to function in the response to UV-induced DNA damage. Although numbers of coding genes and miRNAs have been identified and reported to participate in UV-induced DNA damage response (UV-DDR), the precise role of non-coding RNAs (ncRNAs) in UV-DDR remains largely unknown.

**Methodology/Principal Findings:**

We used high-throughput RNA-sequencing (RNA-Seq) to discover intermediate-size (70–500 nt) ncRNAs (is-ncRNAs) in C. elegans, using the strains of L4 larvae of wild-type (N2), UV-irradiated (N2/UV100) and NER-deficient mutant (*xpa-1*), and 450 novel non-coding transcripts were initially identified. A customized microarray assay was then applied to examine the expression profiles of both novel transcripts and known is-ncRNAs, and 57 UV-DDR-related is-ncRNA candidates showed expression variations at different levels between UV irradiated strains and non- irradiated strains. The top ranked is-ncRNA candidates with expression differences were further validated by qRT-PCR analysis, of them, 8 novel is-ncRNAs were significantly up-regulated after UV irradiation. Knockdown of two novel is-ncRNAs, ncRNA317 and ncRNA415, by RNA interference, resulted in higher UV sensitivity and significantly decreased expression of NER-related genes in *C. elegans*.

**Conclusions/Significance:**

The discovery of above two novel is-ncRNAs in this study indicated the functional roles of is-ncRNAs in the regulation of UV-DDR network, and aided our understanding of the significance of ncRNA involvement in the UV-induced DNA damage response.

## Introduction

Genomic integrity is essential for the survival of the individual and the reproductive success of the species. DNA damage response (DDR) is a functional network combining DNA repair, cellular senescence, cell cycle regulation and apoptosis. The processes work collaboratively to protect organisms against continuous endogenous and environmental stresses [1]. Ultraviolet (UV) irradiation, a known mutagen of major clinical importance in humans, damages DNA through the formation of cyclobutane pyrimidine dimers (CPDs) and 6-4-photoproducts (6–4 PPs) [2]. Nucleotide Excision Repair (NER) is one of the main repair mechanisms involved in the response to UV-induced DNA damage [3]. The importance of this repair mechanism is illustrated clinically by three severe NER-defective syndromes: xeroderma pigmentosum (XP), Cockayne syndrome (CS), and trichothiodystrophy (TTD) [4]. Sufferers of these three syndromes exhibit hypersensitivity to sunlight and a predisposition to skin cancer, indicating that the molecular response to UV light is critical for protecting organisms. Eukaryotic UV-induced DNA damage response (UV-DDR) is a highly conserved network of multi-step processes. UV-induced DNA damage also triggers several other molecular responses such as cell cycle arrest and apoptosis [5]–[7]. *C. elegans* is a useful model for DNA damage response and repair studies because it is a simple organism sharing many homologs with human DDR genes [8], [9]. Despite a detailed understanding of the roles of many key proteins, the mechanisms regulating the UV-induced DNA damage response are not fully elucidated.

In recent years, much effort has been put into unraveling the non-coding transcriptome. Since the landmark discovery in *C. elegans* of the first miRNAs [10], [11], numerous non-coding RNAs of different sizes have been characterized in various organisms and shown to be involved in diverse biological processes [12]. The functions of small RNAs (<200 nt), in particular miRNAs and other RNAs ranging in size between 15 to 40 nt, have been intensively investigated. A series of miRNAs have been reported to target key genes in the DNA damage response and are themselves regulated transcriptionally and post-transcriptionally [13], [14]. However, large-scale high-throughput transcriptome analyses have shown that a substantial fraction of the transcriptome is constituted of longer ncRNAs (>200 nt). Some of these long transcripts have been defined as functional RNAs [15]. Meanwhile, abundant intermediate-size ncRNAs (is-ncRNAs), with a size range between that of small and long ncRNAs (70–500 nt), have been shown to contribute to the transcriptome of all investigated organisms [16]–[18]. These is-ncRNAs include several well-known classes of ncRNAs (*e.g.* snRNA, snoRNA), as well as some novel transcripts such as PASR (promoter-associated small RNA), TASR (terminator-associated small RNA) and enhancer RNA (eRNA) [19]–[21]. We have previously investigated the functions of the is-ncRNAs, showing that they have a high degree of developmentally-specific expression in *C. elegans* [22] and may play important tissue-specific roles in the development and tumorigenesis of the human brain [23]. However, specific analysis of the involvement of is-ncRNAs in UV-DDR in *C. elegans* has been limited.

Here we report a systematic screening and characterization of a panel of novel is-ncRNAs related to UV-DDR in L4 larvae of *C. elegans* using high-throughput RNA-sequencing (RNA-Seq), microarray assays, and qRT-PCR. We identified 57 UV-DDR-related is-ncRNA candidates, several of which were novel is-ncRNAs whose expression was significantly up-regulated after UV irradiation and which contribute to UV resistance. These results increase our understanding of the role of ncRNA in the regulation of the UV-DDR network.

## Results

### High-throughput Sequencing Revealed Hundreds of Novel Candidate Transcripts

**Figure 1:**
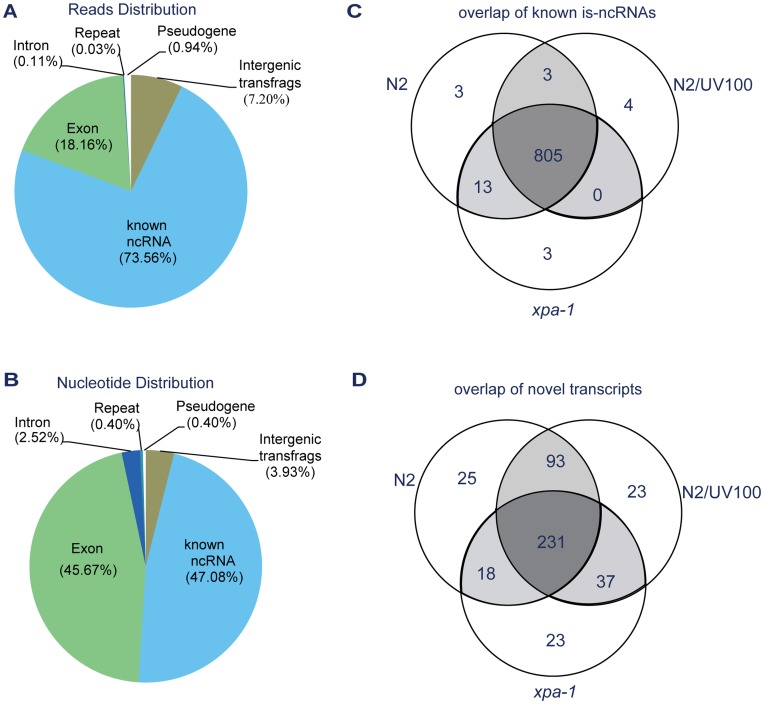
Sequencing data analysis. A and B, Genomic distribution of sequencing data. Average of control, UV-irradiated and *xpa-1* deletion mutant samples. Reads were annotated either as ncRNAs, exons, introns, and pseudogenes and repeats, or as intergenic. (A) Reads distribution. The score for reads that mapped to multiple genomic loci was averaged according to the number of hits. (B) Distribution of genomic nucleotides covered by at least one sequencing read. C and D, Overlap of known is-ncRNAs (E) and novel transcripts (F) in the three RNA-Seq samples.

To discover is-ncRNAs involved in UV-DDR, we treated *C. elegans* with direct UV irradiation. Additionally, since it has been reported that NER is a main pathway to repair UV-induced DNA damage [3], NER deficient mutant was also analyzed to reveal more is-ncRNA candidates in UV-DDR that potentially being modulated by the NER pathway. We performed Solexa RNA-Seq of the transcripts in the size range 70–500 nt from wild-type (N2), UV-irradiated (N2/UV100) and NER-deficient mutant (*xpa-1* deletion) stains. Solexa sequencing generated approximately 12 million reads (36 nt length) from each sample, of which an average of 89.5% reads mapped to the *C. elegans* genome (WS190) with no more than two mismatches (Supplementary Table S1). About two thirds (73.56%) of the reads mapped to known ncRNAs and 7.31% mapped to unannotated regions (Figure 1A). Of all genomic nucleotides covered by mapped reads, 47.08% and 45.67% were annotated as ncRNA and protein-coding genes, respectively, and 6.45% were annotated as intronic or intergenic regions (Figure 1B). Of all known is-ncRNAs, 91.09% was covered by sequencing reads, and some precursor sequences of miRNAs were also detected (see Table 1).

**Table 1:** Detection rate of known ncRNA loci.

ncRNA class	Number of known loci	Detected	Detection Rate (%)
snoRNAs	135	125	92.59
snRNAs	98	87	88.78
tRNAs	630	590	93.65
rRNAs	21	21	100.00
sbRNAs	10	10	100.00
scRNAs	1	1	100.00
SL2 RNAs	8	8	100.00
Sm Y RNAs	1	1	100.00
Uncharacterized is-ncRNAs	61	36	59.02
All interrogated ncRNAs	965	879	91.09
21U-RNAs	5356	30	0.56
miRNAs	139	14	10.07

To determine whether and to what extent the sequencing data might cover the transcription start sites (TSSs) of the detected transcripts, we first made a comparison to annotated is-ncRNA loci. Known is-ncRNAs represented by transfrags with coverage no less than six (597 of 890 annotated is-ncRNAs; WS190) were used for the analysis. For 75% of known is-ncRNAs, the sequencing data covered their annotated TSSs (Supplementary Figure S1). The lack of overlap between sequencing data and TSSs for the remaining 25% of the is-ncRNA loci might reflect post-transcriptional processing of these is-ncRNAs. For these loci, we frequently observed a peak in sequencing density around 40 bp upstream of the annotated TSS. This is reminiscent of previous observations [24], [25] showing that certain subgroups of is-ncRNAs are processed from primary transcripts whose TSSs are located upstream of the 5′terminus (*i.e.,* annotated TSS) of the mature transcript.

We analysed the diversity of expression of known is-ncRNAs and unannotated transcripts. Of the unannotated transcripts, 450 (216 plus strand and 234 minus strand) were represented by transfrags with coverage no less than six, and regarded as novel transcripts. Compared to known is-ncRNAs, novel transcripts were more frequently detected in only one sample. While known is-ncRNAs showed low diversity between samples (Figure 1C), many of the novel transcripts showed sample-specific expression (Figure 1D). These results suggested that the expression of some of novel transcripts was induced under damage stress, and these 450 novel transcripts were used as is-ncRNA candidates for further studies.

### Screening of UV-DDR Related is-ncRNA Candidates

A custom microarray was developed to examine the expression profiles of 568 non-coding transcripts (see Supplementary Table S2), including annotated is-ncRNAs and the identified novel transcripts, in both N2 and *xpa-1* before and after UV irradiation (N2/UV100, *xpa-1*/UV100). To enable comparison of transcript expression among different UV treatment and worm strains, the signal intensities of all arrays were quantile normalized after log2 transformation using the R limma package.

To more accurately identify known and novel transcripts that may be involved in the UV response, we analysed the high-throughput data using the following criteria: (i) the expression level in the UV-irradiated (100 J/m^2^) sample should be at least 1.5-fold higher or lower than that of the untreated sample; (ii) the coefficient of variation of the expression level of the transcripts should be larger than the median of the expression of all detected known is-ncRNAs and novel transcripts; (iii) the change in the expression pattern detected by the microarray should be consistent with the high-throughput RNA-Seq data. Three is-ncRNAs and 44 novel transcripts were selected by these criteria. Normalization of the microarray data using the GRSN method identified a further 10 candidates which fitted the above selection criteria. Most of these transcripts (82%) were novel identified non-coding transcripts, while 10 were is-ncRNAs previously annotated in WS190. Nearly half of the candidates (26 out of 57) were located in intronic regions of protein-coding genes in either strand, eight overlapped with exons, and the remaining candidates were located in intergenic regions. These 57 transcripts (Supplementary Table S3) were regarded as UV-DDR-related is-ncRNA candidates and retained for further analysis.

Comparison of the 47 novel UV-DDR-related candidates with data from previous tiling array assays covering eight different developmental stages or environmental conditions [22], showed that only about 15% (8 out of 47) were detected in the tiling array data. The lack of overlap between the two data set a further indication that these candidates are specifically UV-DDR-related loci that are not transcribed and are not essential during normal development.

### Confirmation of the Involvement of UV-DDR Related is-ncRNA Candidates

**Figure 2:**
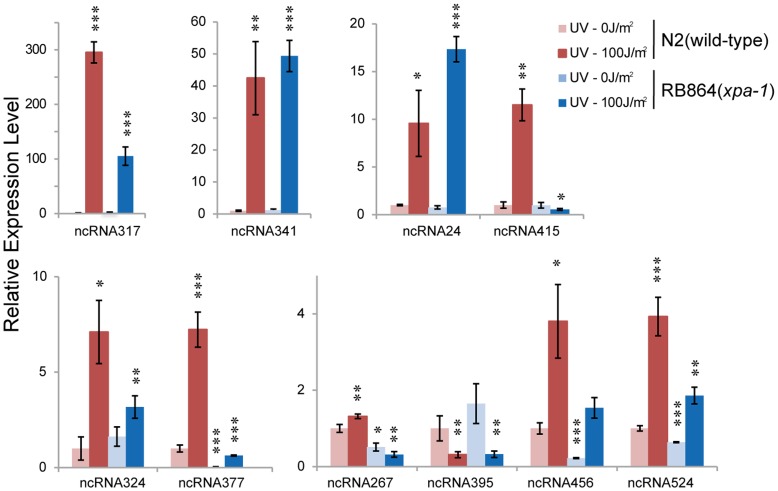
qRT-PCR-validated differential expression levels of UV-DDR-related is-ncRNA candidates. The expression levels of UV-DDR-related is-ncRNA candidates were examined by qRT-PCR in N2 and *xpa-1* deletion mutant before and after UV irradiation (100 J/m^2^). Results were normalized to the expression level of U6 and compared with the level of the wild type without UV irradiation. Data presented are means ± SEM of at least three independent experiments. *: p<0.05, **: p<0.01, ***: p<0.001.

3′-RACE and qRT-PCR were used to validate UV-DDR related is-ncRNA candidates and their differential expression. The ten is-ncRNAs with the highest levels of differential expression (>2-fold) are shown in Figure 2. Among them, ncRNA24 have been annotated as noncoding RNAs with unknown functions (Other noncoding RNAs, i.e. F35E12.11; see Supplementary Table S3), and ncRNA415 and ncRNA317 overlap predictive genes with unknown functions in the latest Wormbase version. However, using RNA-Seq and RACE analysis in our study, we completed more precise 5′and 3′ end of both transcripts.

The overall qRT-PCR results correlated well with the microarray data, giving a Spearman correlation coefficient of 0.636. The two most differentially expressed ncRNA candidates, ncRNA317 and ncRNA341, exhibited dramatic increases in expression induced by UV irradiation in both the wild-type and the *xpa-1* deletion mutant. In contrast to these, ncRNA395 showed significantly lower expression under UV irradiation in both the wild type and the *xpa-1* deletion mutant.

ncRNA415 is representative of another typical expression pattern. This transcript was expressed at wild type level in the *xpa-1* deletion mutant in absence of UV irradiation. After UV irradiation, its expression increased more than 10 fold in the wild type, while no increase was seen in the *xpa-1* deletion mutant, thus, the upregulation of ncRNA415 by UV irradiation appear to dependent on the presence of *xpa-1*. This pattern was in turned contrasted by the novel transcripts ncRNA377, ncRNA456 and ncRNA524, whose expression was considerably reduced in the *xpa-1* deletion mutant. These three transcripts were upregulated after UV irradiation, but showed a weaker UV response in the x*pa-1* deletion mutant.

In addition, ncRNA267 exhibited another expression pattern. In the wild type, ncRNA267 showed a modest upregulation after UV irradiation. While in the *xpa-1* deletion mutant, the expression of ncRNA267 decreased, and this difference was exacerbated after UV-treatment. The opposite responses to UV irradiation in the wild type and the *xpa-1* deletion mutant suggested that a complex interaction including additional factors regulates the expression of ncRNA267.

Taken together, these results confirm that is-ncRNAs show a variety of expression patterns in response to UV irradiation, suggesting that is-ncRNAs are involved in UV-DDR and may have different functional mechanisms.

### Characteristics of UV-DDR Related is-ncRNA Candidates

**Figure 3:**
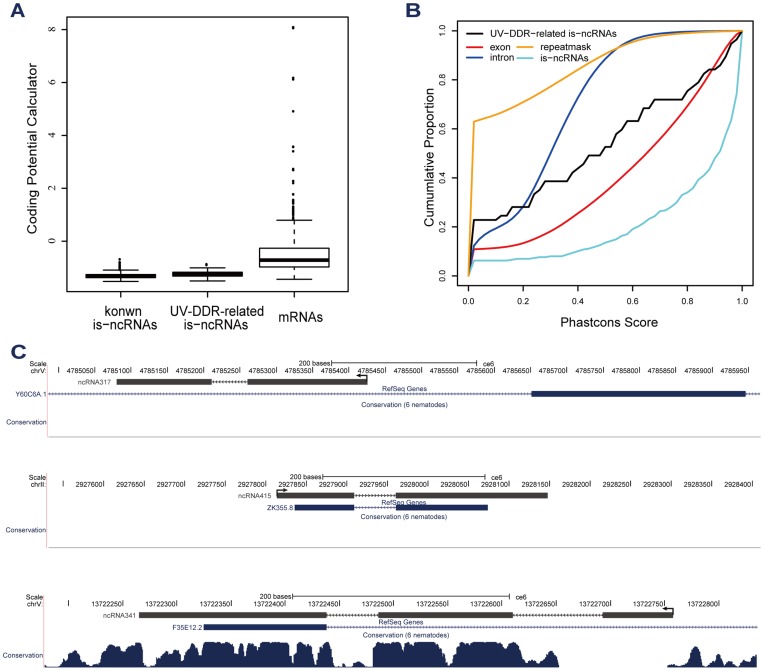
Characteristics of UV-DDR-related is-ncRNAs. A, Coding potential. Coding potentials of known is-ncRNAs, UV-DDR-related is-ncRNAs and 570 randomly selected mRNAs were analysed using CPC. B, Conservation. Conservation of the genomic transcript sequence for repeat sequences, exons, introns, known is-ncRNAs and 57 UV-DDR-related ncRNAs (UV-ncRNA). C, Conservation and location of the three is-ncRNAs. RefSeq genes are in blue and ncRNAs are in gray. Arrows indicate the direction of transcription. The extent of conservation varies with genomic location and is based on PhastCons scores in UCSC. ncRNA317 is located antisense to the intron of protein-coding gene *Y60C6A.1.* ncRNA415 is an intergenic transcript. Based on our sequencing and 3′ RACE results, the 5′- and 3′-end of ncRNA415 was extended 22 nt and 74 nt, respectively, as shown. ncRNA341 comprises 3 exons, which are located at positions equivalent to the deeply conserved regions of RefSeq gene *F35E12.2*.

The protein coding potential of the 57 UV-DDR-related is-ncRNA candidates was analysed using CPC [26], and the coding potential of these transcripts was comparable to that of known is-ncRNAs and lower than that of mRNAs, supporting the annotation of these transcripts as non-coding (Figure 3A). Assessment using PhastCons scores from UCSC showed that the extent of conservation in 57 UV-DDR-related is-ncRNA candidates are lower than for exons of protein-coding genes but higher than for introns and repeat sequences (Figure 3B). These results are in concordance with previous observations in the mouse and human genomes [27], [28]. Several is-ncRNAs, such as ncRNA317 and ncRNA415, showed little conservation and have no homologs or orthologs in other *Caenorhabditis* species, suggesting that these is-ncRNAs are likely *C. elegans-*specific. The conserved regions of other is-ncRNAs overlap with CDSs, for example the conserved regions of ncRNA341 are located at positions equivalent to exon 1 and intron 1 of gene F35E12.2 (Figure 3C). Several of these UV-induced is-ncRNAs appear to undergo splicing, a phenomenon which has hardly been characterized before for this size of ncRNAs expect several well-known tRNAs (Figure 3C). The splicing followed a GU-AG pattern, suggesting that the splicing machinery may be the same as that for mRNAs. To determine whether the UV-induced transcripts were polyadenylated, we compared the genomic regions of these transcripts with 3P data [29], and 50% of the ten most differentially expressed ncRNAs were polyadenylated (Supplementary Figure S2). It is suggested that some *C. elegans* is-ncRNAs can be processed post-transcriptionally through splicing and polyadenylation similarly to what has been found for mammalian long ncRNAs [30].

### Three is-ncRNAs Contributing to UV Resistance

To investigate the functional roles of UV-DDR-related is-ncRNA candidates, we determined UV sensitivity and NER-related gene expression after targeted knockdown of specific transcripts. ncRNA317, ncRNA341 and ncRNA415 were selected for study due to their strong response to UV irradiation and different expression patterns. After knockdown of the selected ncRNAs using RNAi via feeding, we studied the UV survival in L4 larvae and the NER-related gene expression.

**Figure 4:**
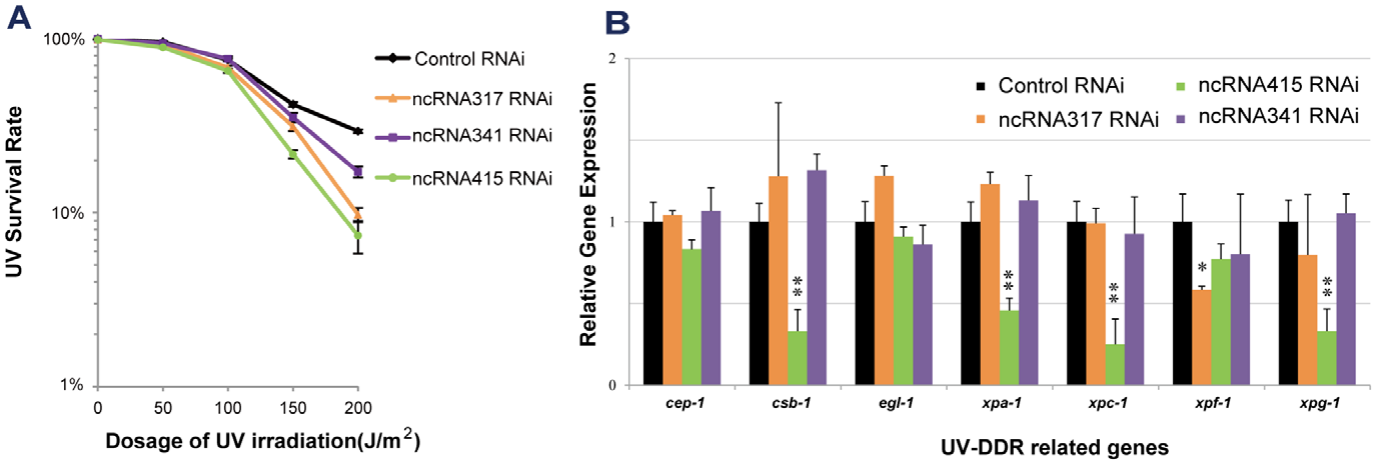
Three is-ncRNAs contributing to UV resistance. Candidate ncRNAs were knocked down by RNAi via feeding in wild-type worms. A, L4 larval survival following UV irradiation. Worms of L4 stage larvae were exposed to UV irradiation (0, 50, 100, 150, or 200 J/m^2^). Worm survival was determined about 24 h after UV treatment. Each point represents the mean of three independent experiments, each performed in duplicate (typically, n>60). Error bars denote the SEM. B, Effects of target is-ncRNAs knockdown on UV-DDR-related genes. Quantitative RT-PCR was used to detect the relative expression level of UV-DDR-related genes in RNAi-treated adult worms. Error bars show the mean ± SEM of at least three independent experiments. *: p<0.05, **: p<0.01, ***: p<0.001.

A modified UV survival assay similar to that of Lans *et al.* [31] was set up to measure survival of UV-irradiated L4 larvae. Survival was scored by determining the percentage of animals capable of growing to adulthood. In the absence of UV irradiation, these RNAi-treated worms barely showed any developmental arrest, suggesting that developmental arrest is a suitable marker for evaluating UV survival. RNAi-mediated knockdown of ncRNA317 and ncRNA415 resulted in markedly increased UV-sensitivity at all tested dosages (p-value<0.05, paired *t*-test; Figure 4A), indicating that these two is-ncRNAs make a strong contribution to UV resistance. Knockdown of ncRNA341 led to a slight but significant enhancement in UV sensitivity and in developmental arrest in response to a relatively high UV dosage in L4 larvae (150 and 200 J/m^2^; P-value <0.05; Figure 4A).

To investigate the effects of candidate is-ncRNAs knockdown on the expression levels of UV-DDR related genes, we used qRT-PCR to detect the relative expression of UV-DDR genes in RNAi-treated worms. Some NER genes exhibited lower expression levels in both ncRNA317 and ncRNA415 RNAi-treated worms compared with control worms, while the expression level of *egl-1* which is a key activator of apoptosis in *C. elegans* [32] was stable, suggesting that the contribution of these two is-ncRNAs in the UV response is probably via participation in the NER pathway rather than in apoptosis (Figure 4B). When ncRNA317 was knocked down in L4 larvae, significantly lower expression was observed only for gene *xpf-1*; however, when ncRNA415 was knocked down, there was a general decrease in the expression of a series of NER genes, including *csb-1*, *xpa-1*, *xpc-1* and *xpg-1*. This difference in the genes knocked down by ncRNA317 and ncRNA415 suggests that the functional mechanisms of these two is-ncRNAs may differ. Knockdown of ncRNA341 did not influence the expression levels of UV-DDR related genes, except that *xpf-1* showed a mild decrease in expression (Figure 4B). This may because ncRNA341 contributes to UV resistance via pathways other than Nuclear Excision Repair. The increases in UV sensitivity and effects on NER genes observed here provide further confirmation that these three ncRNAs are implicated in the UV-induced DNA damage response.

## Discussion

Although many UV-DDR-related coding genes have been identified, the mechanism underlying the UV-induced DNA damage response remains unclear. Recent evidences have suggested that several miRNAs [14] and a few long ncRNAs (e.g. ncRNA_CCND1_) [33] play important roles in the regulation of DNA damage responses. Systematic discovery and identification of is-ncRNAs involved in UV-DDR will thus complement and extend our knowledge of the DNA damage response network.

In the present study, we have analysed the intermediate-size (70–500 nt) transcriptome of wild-type N2, UV-irradiated N2 and *xpa-1*-deficient strain using RNA-Seq technology. More than 90% of known is-ncRNAs were detected here, and the remaining is-ncRNAs were probably not detected due to low levels of expression or stage-specific expression. After filtering, 450 novel transcripts were identified. The expressional complexity of these novel transcripts in the RNA-Seq samples suggested them possible functional.

In agreement with our observations from RNA-Seq data, the majority of changes in expression level for non-coding transcripts detected in the microarray were contributed by novel transcripts. The differential expression of these transcripts may indicate that they play important roles in the UV response in *C. elegans*.

Microarray revealed 57 UV-DDR-related is-ncRNA candidates that were differentially expressed after UV irradiation or in the *xpa-1* deletion mutant. Analysis of our microarray and qRT-PCR data showed expression patterns typical of these candidates. NcRNA317 was present at low abundance under control conditions, but showed high expression levels after UV treatment. This response pattern resembles that of lncRNAs upstream of CCND1, which are generally present at a level of even less than two copies per cell, but once induced in response to DNA damage are present at higher levels [33]. Several miRNAs have been reported to be significantly up-regulated after UV irradiation and to participate in the DNA damage response [14].

In addition to the up-regulation of the transcripts in response to UV radiation, some transcripts were down-regulated, for example ncRNA395. miRNAs belonging to the let-7 family, most of which have been shown to play a significant role in the radiation response [34], provide a typical example of functional down-regulation after UV irradiation. It is also possible that some of the decreases in expression recorded here might result from increased RNA degradation or damage-induced blocks in transcription [35], following exposure to UV.

In the top-ten differential expressed is-ncRNAs, ncRNA317 and ncRNA415 overlapped with the transcripts of gene Y60C6A.2 and ZK355.8, respectively. Y60C6A.2 has not been annotated in WS190 until WS215, and ZK355.8 is a predictive gene with unknown function. We performed the conservation analysis and coding potential assessment. The little conservation and low coding potential score supported the annotation of ncRNA317 and ncRNA415 as non-coding RNAs. Specially, ncRNA317 was found to be not polyadenylated according to 3P data [29] and our study. Furthermore, due to our RNA-Seq and RACE analyses, we updated the gene annotation and gave more precise 5′and 3′end of both transcripts. The 5′- and 3′-end of ncRNA415 was extended 22 nt and 74 nt, respectively, and the 5′-of ncRNA317 was extended 8 nt. Thus, we are inclined to think that transcripts we annotated are the real, complete transcripts. The rest of the top ncRNAs were all analyzed as described for ncRNA317 and ncRNA415. Except for a known is-ncRNA (ncRNA24, F35E12.11) and ncRNA341 which comprises 3 exons and overlaps partly with exon1 of RefSeq gene F35E12.2, other transcripts were not found to have any splicing and overlap with known genes.

We have successfully demonstrated the contribution of ncRNA317 and ncRNA415 to UV survival. Interestingly, the effect of RNAi on expression levels of different UV-DDR-related genes indicates that these ncRNAs affect UV-DDR by diverse mechanisms. It is well known that *csb-1* and *xpc-1* initiate transcription-coupled repair (TCR) and global genomic repair (GGR), respectively, in order to recognize DNA damage, and that *xpa-1* then verifies the damage and subsequently assembles other NER factors including *xpf-1* and *xpg-1* to execute the repair functions [31]. The decrease in *xpf-1* expression in ncRNA317 RNAi-treated worms suggests that the contribution of ncRNA317 to UV sensitivity may owe to a regulatory role of this transcript in the NER repair phase. ncRNA415, whose knockdown resulted in lower expression levels of both *csb-1* and *xpc-1*, may be involved in the regulation of DNA damage-sensing mechanisms, which are the early stages of the NER pathway. In addition, it should be noted that i) the UV-induced expression of ncRNA415 was dependent on the presence of *xpa-1* and ii) *xpa-1* and *xpg-1* showed reduced expression in ncRNA415-knockdown worms. We thus hypothesize that ncRNA415 cooperates with *xpa-1*, and that this cooperation is required for the stability of *xpa-1* and its function in UV-DDR. Unlike knockdown of ncRNA317 and ncRNA415, knockdown of ncRNA341 did not result in significant differential expression of NER genes. Therefore, if ncRNA341 has any role in UV-DDR, it is likely to be exerted in some other way than through the NER pathway. For example, hsa-miR-34 and hsa-miR-16 are induced by UV-irradiation in humans, and trigger G1 cell cycle arrest in response to DNA damage [36].

Some longer transcripts can be processed to yield small RNAs such as miRNAs, piRNAs, and other less well-characterized classes of small transcripts [15]. Hence, synthesis of miRNAs from is-ncRNAs may be another mechanism by which is-ncRNAs are involved in UV-DDR. In addition, some of the longer UV-DDR-related transcripts (*e.g.* ncRNA415) are apparently spliced and polyadenylated, similar to lincRNAs (long intergenic ncRNAs) in the mammalian genome [30].

In conclusion, this work identified 450 novel intermediate-size transcripts in *C. elegans* by RNA-Seq, and the microarray analysis revealed 57 is-ncRNA candidates with considerable difference in expression between the wild-type and a NER-deficient mutant (*xpa-1* deletion) before and after UV irradiation, indicating that the complexity of the *C. elegans* UV response include hitherto unknown non-coding elements. Our results not only identify a number of is-ncRNAs that will be interesting subjects for future study, but also provide evidence that some of these novel transcripts are intrinsically functional. Importantly, the contribution of ncRNA317 and ncRNA415, two novel is-ncRNAs, to UV resistance and their involvement in the UV-DDR were demonstrated. The novel is-ncRNAs reported in this study represent an important contribution to our understanding of the significance of ncRNA involvement in the UV-induced DNA damage response.

## Materials and Methods

### Nematode Strain Preparation

Two *C. elegans* strains, N2 (wild type) and RB864 (*xpa-1* deletion, NER pathway deficient) were used. The RB864 strain was backcrossed five times with the N2 strain to obtain the same genomic background as the N2 strain [37]. Simple deletion locus-specific PCR was used to track and maintain the deletion alleles throughout the backcrossing process. L4 larval stage worms were obtained by growing synchronized L1s (L1 starved) worms on NGM plates seeded with OP50 at 20°C for 44 hours [24], [38].

### UV Irradiation

To determine an appropriate dosage of UV irradiation, increasing doses of UV irradiation from 0 to 140 J/m^2^ at intervals of 20 J/m^2^ (UVC 254 nm; UVP CX-2000) were applied to *C. elegans* strain N2 at the L4 developmental stage. NER-related genes (listed in Table 2) were amplified by quantitative RT-PCR (primers listed in Supplementary Table S4), and the up-regulation of gene expression was evaluated. The optimum dosage of UV treatment was determined to be 100 J/m^2^, in which condition all of the detected genes were up regulated more than 2 fold-change (Supplementary Figure S3), and this dose was applied in all subsequent experiments.

**Table 2:** Summary of mRNA genes analysed by qRT-PCR.

Name	ID	*H. sapiens* Orthologs	Function (in *C. elegans*)	Reference
*cep-1*	F52B5.5	p53	Regulatory factor in DDR	[44], [45]
*csb-1*	F53H4.1	CSB	Functions in Transcription Coupled Repair (TCR)	[31], [46]
*egl-1*	F23B12.9	BH3	An upstream activator in general apoptosis	[32], [45]
*xpa-1*	K07G5.2	XPA	Verifies damage and assembles the repair machinery.	[47]
*xpc-1*	Y76B12C.2	XPC	Functions in Global Genome Repair (GGR)	[31]
*xpf-1*	C47D12.8	XPF	DNA incision at 5′ ends	[48]
*xpg-1*	F57B10.6	XPG	DNA incision at 3′ ends	[9]

L4 worms were washed three times with 0.1 M NaCl solution to remove bacterial contaminants, transferred to new unseeded plates and exposed to UV (100 J/m_­_^2^). Treated worms were allowed to recover for 3 h before extracting RNA.

### RNA Extraction

Total RNA was extracted from control and UV-irradiated worms with Trizol reagent (Invitrogen) according to the manufacturer’s instructions. Contaminant DNA was removed with DNase I (Fermentas).

### Solexa Sequencing

A modified sample preparation strategy was developed for the is-ncRNAs Solexa sequencing. Intermediate-size transcripts (70–500 nt) were size-fractionated by excising the band region corresponding to appropriate RNA standard markers from a 6% denaturing urea PAGE gel. After extracting from the gel, rRNAs were removed using MicrobExpress kits (Ambion) with specifically designed probes. The shorter fractions (70–200 nt) were dephosphorylated with CIAP (Fermentas), ligated to a 3′-adaptor (3′AD, 5-p-UCGUAUGCCGUCUUCUGCUUG-idT-3) using T4 RNA ligase (Promega) and reverse-transcribed with a universal primer (3′RT, CAAGCAGAAGACGGCATACGA), while the longer fractions (200–500 nt) were directly reverse transcribed with a random primer (5-CAAGCAGAAGACGGCATACGANNNNNN-3). A double-stranded cDNA library was constructed with a SMART™ cDNA Library Construction Kit (Clontech) and sequenced using standard single end Solexa sequencing.

The sequencing reads were mapped to the *C. elegans* genome (WS190) using Bowtie [39], allowing no more than two mismatches. Mapped reads were merged to wiggle format files by SAMtools and customized scripts. The transfrags in wiggle files were selected as candidates. The base coverage of detected transfrags was used to filter out novel transcripts. Transfrags of low coverage (coverage<6), shorter length (<36 nt) or overlapped with annotated regions were removed. Transfrags shorter than 70 nt were extended downstream to 70 nt for microarray probe design. The remaining 450 novel transcripts were regarded as is-ncRNA candidates’ tags and subjected to microarray analysis. Raw data and processed wig files can be accessed from GEO via GSE37063.

### Non-coding Transcripts Microarray

All the 193 is-ncRNAs annotated in WS190 (tRNAs and rRNAs excepted) and 450 novel transcripts identified by Solexa sequencing (extended from detected 5′ ends to 70 nt according to the minimal length for size-fraction) were combined (643 in total) for probe design using OligoArray 2.0 [40]. Oligo length was set to between 38 and 42 nt, and the predicted melting temperature confined to a range from 79 to 90°C. Using these criteria, 568 probes were successfully designed. In addition, 65 negative control probes were selected from the human genome with the same criteria.

RNA samples were prepared according to a previously published method [41] with some modifications. The poly (A) fraction was first isolated from total RNA using a Poly (A) Purist™ MAG Kit (Ambion). After removal of rRNAs, the non-poly(A) fraction was poly(A) tailed using a Poly(A) tailing Kit (Ambion) and combined with the poly(A) fraction for first strand synthesis of cDNA with an oligo (dT) primer containing a T7 RNA polymerase promoter. After second-strand synthesis an in vitro transcription was carried out using T7 RNA polymerase to amplify the antisense RNAs (aRNAs), which were then directly labeled with Cy5 for hybridization.

The signal intensity of all arrays was quantile normalized after log2 transformation using the R limma package to compare transcript expression among different UV irradiation and worm strains. The coefficient of variation of the expression level of the transcripts among different samples detected in the RNA-Seq or microarray was calculated for screening.

### 3′ RACE

Total RNA was ligated with 3′AD and reverse transcribed with 3′RT primer. The target regions were amplified by PCR (Takara) with transcript-specific primers and 3′RT primer (primers listed in Supplementary Table S1). The PCR products were analysed on a 6% native PAGE gel and the candidate bands were recovered for sequencing.

### SYBR Green-based Quantitative RT-PCR

All primers were designed using Primer Premier 5 based on published sequence data from Wormbase and our 3′ RACE sequencing results. The specificity of primer sequences was further confirmed using primer–BLAST (NCBI) (primers listed in Supplementary Table S1).

Quantitative RT-PCR reactions were carried out in TransScript™ II Green One-Step qRT-PCR Super Mix (TransGen) using a CFX96™ Real-Time PCR Detection System (Bio-Rad). Cycling conditions were 50°C for 5 min (for reverse transcription) and 94°C for 30 s, followed by 40 cycles of 94°C for 5 s, 60°C for 15 s, and 72°C for 10 s. The expression levels were normalized to *act-1* for coding genes and to U6 snRNA for non-coding transcripts, and the relative expression was calculated as 2^−ΔΔCt^. Nuclease-free water was used as the template in the NTC (no template control).

### RNAi

RNAi was carried out by feeding worms with *E. coli* HT115 (DE3) carrying plasmid L4440, expressing a dsRNA fragment of inserted DNA sequences of targeted transcripts. Plasmid L4440 target unc-22 was chosen as a positive control for RNAi since it results in distinct post-embryonic phenotype-uncoordinated movements (Unc), and an empty vector was used as a negative control. Bacterial cells were applied to RNAi plates (NGM plates supplemented with 25 µg/ml Carbenicillin and 1 mM IPTG) [42], and incubated at room temperature for more than 20 h before placing the worms on the plates [43]. Under optimized conditions, feeding L1s worms for at least 60 h with plasmid target unc-22, resulted in 98% of the worms showing uncoordinated movements. The expression level of target noncoding RNAs was examined by qRT-PCR after RNAi feeding for 64 hrs, which is the time for the survival statistics, and strong blocking effect was observed for the three is-ncRNAs (Supplementary Figure S4).

### UV Survival Assay for L4 Larvae

UV Survival assay was applied to measure the UV sensitivity of L4 larvae. Eggs were first collected from gravid adult animals by hypochlorite treatment, and then synchronized to L1s (L1 starved) stage. The synchronized L1s (L1 starved) worms were then transferred to prepared plates pre-seeded with *E. coli* HT115 (DE3) bacteria for RNAi, and incubated at 20°C for 44 hours to L4 stage. The L4 worms were transferred to unseeded plates, UV-irradiated from 0 to 200 J/m^2^ at intervals of 50 J/m^2^ (the same as in the experiments described above) and then placed on fresh RNAi bacteria-seeded plates. Animals that developed beyond the L4 stage (survivors) and animals with arrested development were counted to determine the survival percentage [31]. Statistical analysis was performed using *t*-tests.

## Supporting Information

Figure S1 **Detection of transcription start sites.** (A) Two peaks are present in the distribution of the distance (in nt) of the 5′ terminus from the annotated TSS; one at the annotated TSS loci (B) and one at the UM2 loci (∼30 bp upstream) (C).
(PDF)

Figure S2 **The genomic landscape of five polyadenylated noncoding RNAs with tracks for 3P data and RNA-Seq in UCSC genome browser.** For L4_PA track, which represent the 3P data, the blue and orange track indicate the polyadenylated value on the plus and minus strand, respectively. The heavier the colour is, the larger the value is.
(PDF)

Figure S3 **Relative expression level of NER-related genes after UV irradiation.** L4 stage worms were exposed to UV irradiation. All target genes exhibited significantly higher expression levels (p<0.01) after UV irradiation at a dosage of 100 J/m^2^. Results are normalized to the expression level of *act-1* and compared to the expression levels in wild-type not exposed to UV. Error bars denote the mean ± SEM of three independent experiments.
(PDF)

Figure S4 **qRT-PCR-validated the level of knock-down.** The expression levels of target ncRNA were examined by qRT-PCR in RNAi-treated worms. Results were normalized to the expression level of U6 and compared with the level of the RNAi-Empty. Data presented are means ± SEM of at least three independent experiments.
(PDF)

Table S1 **Mapping of sequencing reads to the reference genome.**
(PDF)

Table S2 **The probe sequences and detected expression levels of 568 non-coding transcripts in the RNA-seq and microarray.**
(XLS)

Table S3 **The detected expression levels of 57 UV-DDR-related is-ncRNA candidates in the RNA-Seq and microarray.**
(XLS)

Table S4 **Oligos used in this work.**
(PDF)
