# Supplementary figures and images for: Identification of Intermediate-Size Non-Coding RNAs Involved in the UV-Induced DNA Damage Response in C. elegans
Source: PLoS One. 2012 Nov 7;7(11):e48066. doi: 10.1371/journal.pone.0048066 (PMC3492359; doi:10.1371/journal.pone.0048066)

**A**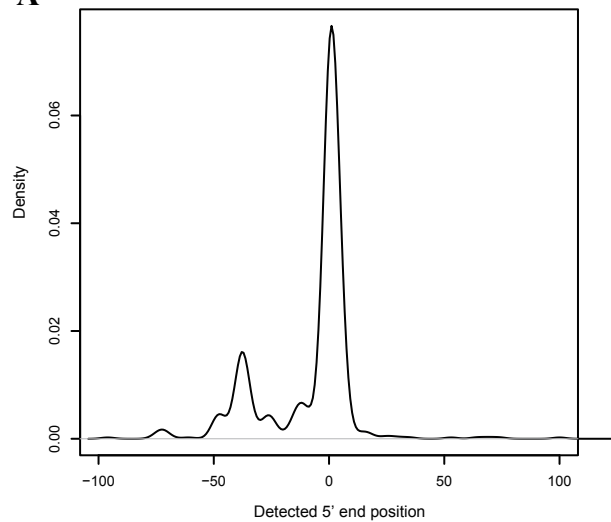**B**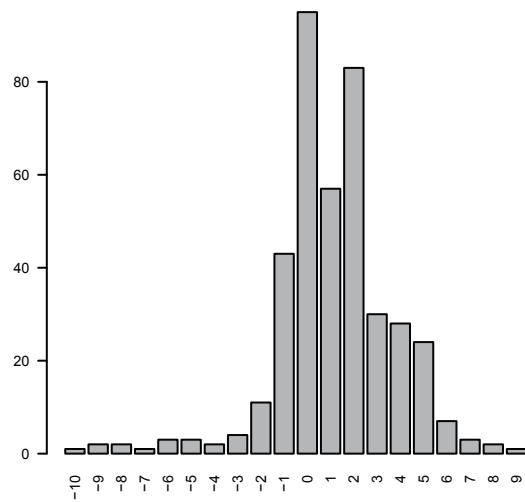**C**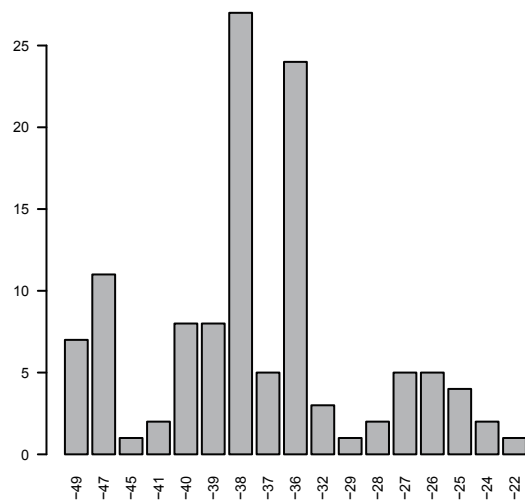

Supplement: Figure S1 — Detection of transcription start sites. (A) Two peaks are present in the distribution of the distance (in nt) of the 5′ terminus from the annotated TSS; one at the annotated TSS loci (B) and one at the UM2 loci (∼30 bp upstream) (C). (PDF) [file pone.0048066.s001.pdf]

A ncRNA524

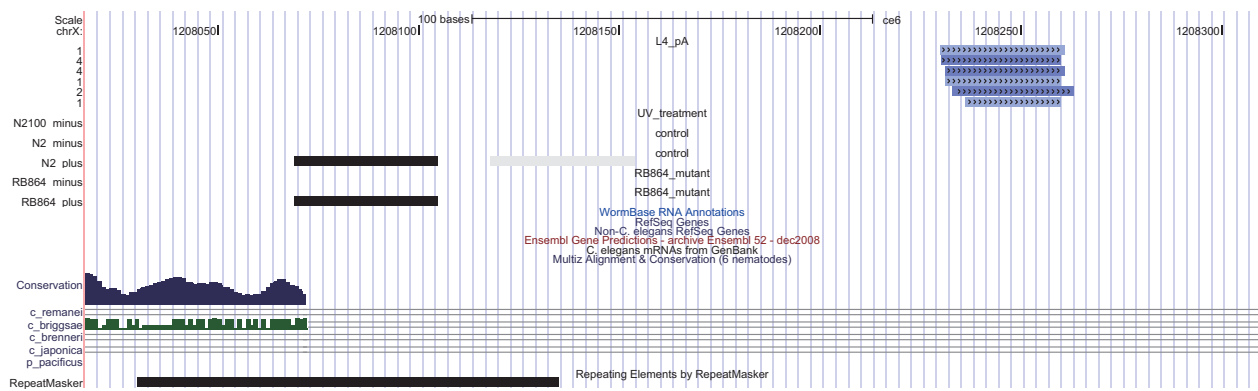

B ncRNA456

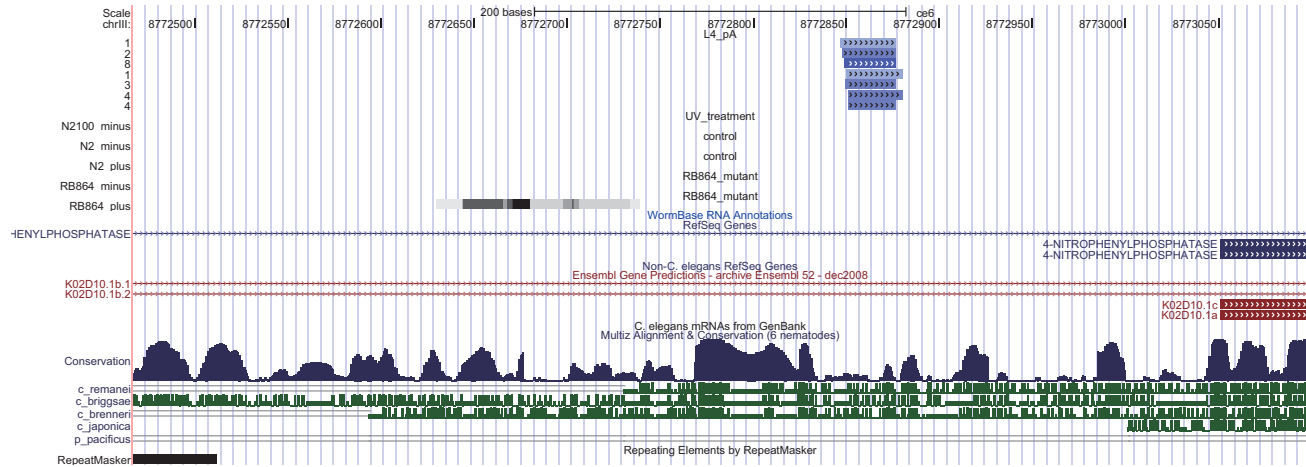

C ncRNA415

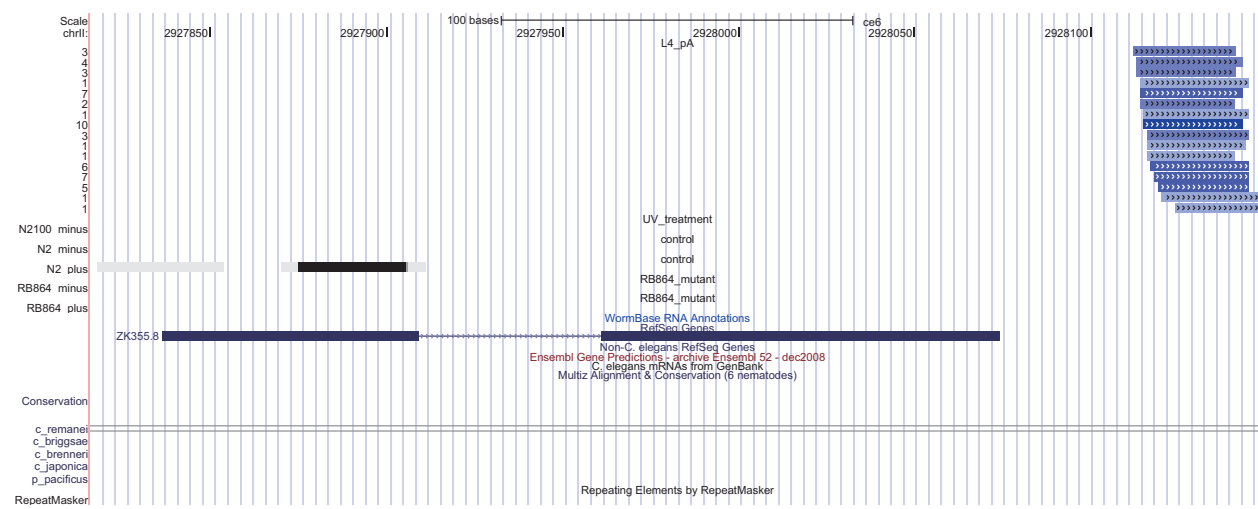

D ncRNA377

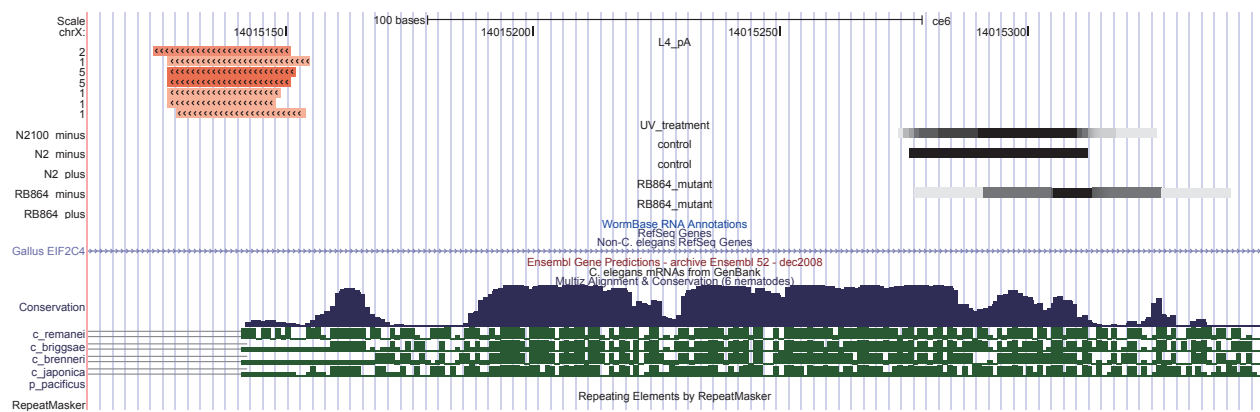

E ncRNA324

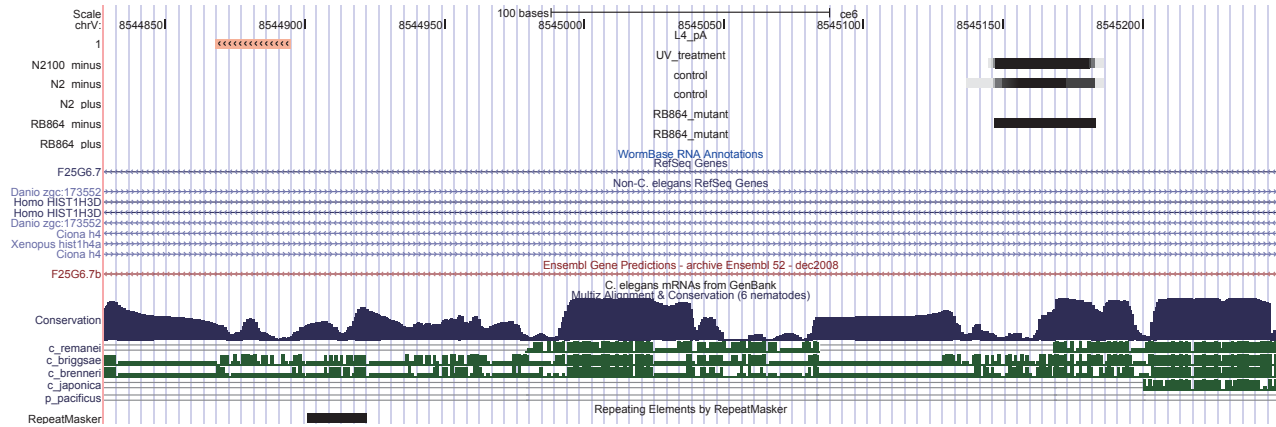

Supplement: Figure S2 — The genomic landscape of five polyadenylated noncoding RNAs with tracks for 3P data and RNA-Seq in UCSC genome browser. For L4_PA track, which represent the 3P data, the blue and orange track indicate the polyadenylated value on the plus and minus strand, respectively. The heavier the colour is, the larger the value is. (PDF) [file pone.0048066.s002.pdf]

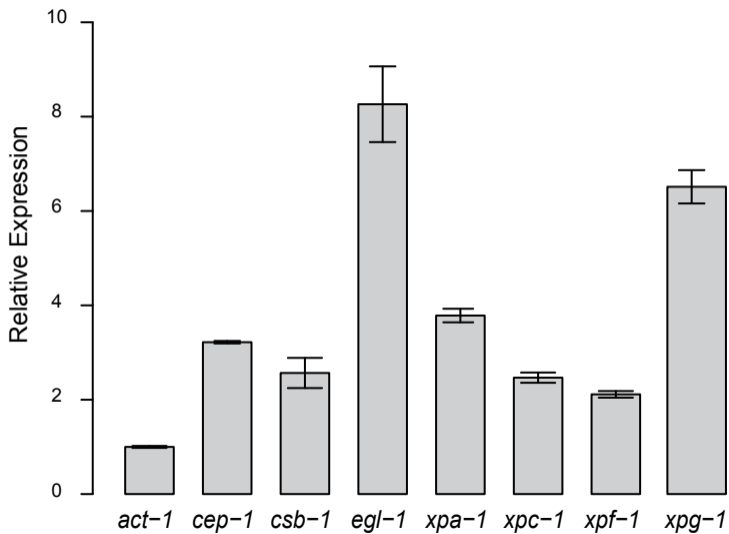

Supplement: Figure S3 — Relative expression level of NER-related genes after UV irradiation. L4 stage worms were exposed to UV irradiation. All target genes exhibited significantly higher expression levels (p<0.01) after UV irradiation at a dosage of 100 J/m2. Results are normalized to the expression level of act-1 and compared to the expression levels in wild-type not exposed to UV. Error bars denote the mean ± SEM of three independent experiments. (PDF) [file pone.0048066.s003.pdf]

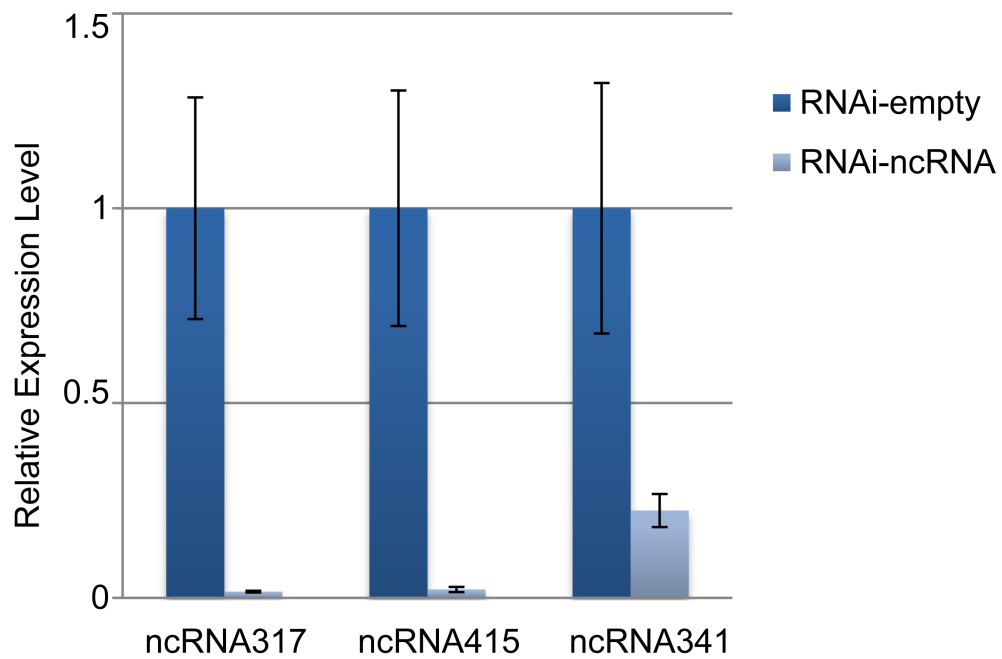

Supplement: Figure S4 — qRT-PCR-validated the level of knock-down. The expression levels of target ncRNA were examined by qRT-PCR in RNAi-treated worms. Results were normalized to the expression level of U6 and compared with the level of the RNAi-Empty. Data presented are means ± SEM of at least three independent experiments. (PDF) [file pone.0048066.s004.pdf]
