# Supplementary material for: Identification of Intermediate-Size Non-Coding RNAs Involved in the UV-Induced DNA Damage Response in C. elegans
Source: PLoS One. 2012 Nov 7;7(11):e48066. doi: 10.1371/journal.pone.0048066 (PMC3492359; doi:10.1371/journal.pone.0048066)
Supplement: Table S1 — Mapping of sequencing reads to the reference genome. (PDF) [file pone.0048066.s005.pdf]

Supplementary Table S1: Mapping of sequencing reads to the reference genome

Sample	Read Counts	Mapped	Unique	Unmapped	Repeat	%Mapped	%Unmapped	%Repeat
N2	11971290	10611349	6220669	1216632	143309	88.6	10.2	1.2
N2/UV100	12531564	10825836	6894756	1471566	234162	86.4	11.7	1.9
xpa-1	13698599	12762783	9772734	867006	68810	93.2	6.3	0.5
**Total**	**38201453**	**34199968**	**22888159**	**3555204**	**446281**	**89.5**	**9.3**	**1.2**
