# Supplementary material for: Identification of Intermediate-Size Non-Coding RNAs Involved in the UV-Induced DNA Damage Response in C. elegans
Source: PLoS One. 2012 Nov 7;7(11):e48066. doi: 10.1371/journal.pone.0048066 (PMC3492359; doi:10.1371/journal.pone.0048066)
Supplement: Table S4 — Oligos used in this work. (PDF) [file pone.0048066.s008.pdf]

Supplementary Table S4: Oligos used in this work

**Primers used for *xpa-1* deletion detection**	
RB864-F	GTCAATGGCAATTTGCTAGTAT
RB865-M	CTGTCCATCAAACCTTTGTCGC
RB866-R	CTGAATTTCTGCGCATGCTT
**Gene-specific primers used for qRT-PCR**	
act-1-F	GCTATGTTCCAGCCATCCTTCT
act-1-R	CAGTGTTGGCGTACAAGTCCTTA
cep-1-F	TACAATGATGCACAGAACTCCACC
cep-1-R	GTACAGCGACTTCTCTTCATCGAC
csb-1-F	AGTTCTTCAATGGTGTGCCTGG
csb-1-R	TCTTCTTCACTGGCGACAGCA
egl-1-F	ATGCTCACCTTTGCCTCAACC
egl-1-R	ACGAGGAGAAGTCCTGAGACGA
xpa-1-F	AAAGAGCTTCGTGAGCAATCCA
xpa-1-R	CGTGAGTATGAGGTCTTCCGAAAT
xpc-1-F	CAAAGGAATGCTGCGTCTCG
xpc-1-R	CATGCATCGGTGTGATCTGTTC
xpg-1-F	TGCCTACCTTCATCCCATCGT
xpg-1-R	TCTTCGTTGTGCTGTTTGCG
xpf-1-F	TCGAGCCATATCCCTGAAAGC
xpf-1-R	GATGACAGTGGGATTCGGTTTC
**ncRNA-specific primers used for 3' RACE and qRT-PCR**	
U6-F	GGAACAATACAGAGAAGATTAGC
U6-R	GGAACGCTTCACGAATTTGC
ncRNA24-F	ATGCTCTTCACAATGCTTCACAG
ncRNA24-R	CAAGTTGTTGTTGATGGGAGAAAC
ncRNA267-F	GTTCATGGAGAGTACCACGCATC
ncRNA267-R	ATAGCCGTTTCAAAACACTAACCTC
ncRNA317-F	CCACTGCATTTGACAAGGACCTC
ncRNA317-R	GCGGTTTCCAGAGTTGACACAC
ncRNA324-F	TTGTGCTACTGATGTGTCTGC
ncRNA324-R	TCGGATTATCATTCCTCTGCTC
ncRNA341-F	CAAATAGTGATTGAATAGCTGTAGC
ncRNA341-R	CGGTCGCTGAAATCCGAACAT
ncRNA377-F	CTCTTATCCATCATATCAAACTTCG
ncRNA377-R	GCGTGTGCCAATCATCAAG
ncRNA395-F	GCAGTGTCGAGAAGATGGTATC
ncRNA395-R	ACAGAAAATCCTTGCACATCCC
ncRNA415-F	AGAACACATTATGGACCCCCAAGGC
ncRNA415-R	TGCTTCTCAACGGAGCGCGA
ncRNA456-F	TCAGTTTTGACTCTTCCAAGATTTC
ncRNA456-R	GTGAAAAGAATTGATGGGAGATTGC
ncRNA524-F	GGAGGCTTGGCACATTGGTAG
ncRNA524-R	ATGACGAGTAGAAAATGTTGAACCG
**Primers used for RNAi experiments**	
ncRNA317-F-RNAi	CCGCTCGAGCGGATGCTTTGCAAAATTTTCATTCCA
ncRNA377-R-RNAi	GGGGTACCCCAGGATAAAATCCACTGTCGCGT
ncRNA341-F-RNAi	CCCAAGCTTGGGTGTGTTTACAAATAGTGATTGAA
ncRNA341-R-RNAi	GGGGTACCCCAAGCAAATTACAATAATCATCGA
